# Intrinsic Migratory Properties of Cultured Schwann Cells Based on Single-Cell Migration Assay

**DOI:** 10.1371/journal.pone.0051824

**Published:** 2012-12-14

**Authors:** Ying Wang, Hong-Lin Teng, Zhi-hui Huang

**Affiliations:** 1 School of Laboratory Medicine and Life Science, Wenzhou Medical College, Wenzhou, Zhejiang, China; 2 Institute of Hypoxia Medicine and Institute of Neuroscience, Wenzhou Medical College, Wenzhou, Zhejiang, China; 3 Department of Spine Surgery, the First Affiliated Hospital of Wenzhou Medical College, Wenzhou, Zhejiang, China; Universidade Federal do Rio de Janeiro, Brazil

## Abstract

The migration of Schwann cells is critical for development of peripheral nervous system and is essential for regeneration and remyelination after nerve injury. Although several factors have been identified to regulate Schwann cell migration, intrinsic migratory properties of Schwann cells remain elusive. In this study, based on time-lapse imaging of single isolated Schwann cells, we examined the intrinsic migratory properties of Schwann cells and the molecular cytoskeletal machinery of soma translocation during migration. We found that cultured Schwann cells displayed three motile phenotypes, which could transform into each other spontaneously during their migration. Local disruption of F-actin polymerization at leading front by a Cytochalasin D or Latrunculin A gradient induced collapse of leading front, and then inhibited soma translocation. Moreover, in migrating Schwann cells, myosin II activity displayed a polarized distribution, with the leading process exhibiting higher expression than the soma and trailing process. Decreasing this front-to-rear difference of myosin II activity by frontal application of a ML-7 or BDM (myosin II inhibitors) gradient induced the collapse of leading front and reversed soma translocation, whereas, increasing this front-to-rear difference of myosin II activity by rear application of a ML-7 or BDM gradient or frontal application of a Caly (myosin II activator) gradient accelerated soma translocation. Taken together, these results suggest that during migration, Schwann cells display malleable motile phenotypes and the extension of leading front dependent on F-actin polymerization pulls soma forward translocation mediated by myosin II activity.

## Introduction

Myelinating glial cells provide an insulating sheath around axons which is required for the rapid propagation of action potentials and the normal function of nervous systems [1], [2]. Schwann cells are the major myelinating glial populations in peripheral nervous system. The formation of peripheral myelin by Schwann cells can be divided into three major stages: proliferative, premyelinating and myelinating stages. The proliferative stage is characterized by proliferation and migration of premyelinating Schwann cells [3]–[6]. During development, Schwann cells arise from trunk neural crest cells, proliferate, and migrate into peripheral nerve. Finally, Schwann cells associate with a single axon, ensheath individual axon and eventually form the myelin sheath [6], [7]. Therefore, Schwann cell migration is critical for development of peripheral nervous system.

Schwann cell migration is also essential for the regeneration and remyelination after nerve injury [8]–[14]. In axonal regeneration after peripheral nerve injury, Schwann cells proliferate, migrate from the proximal and distal part of the transected nerve, and eventually form a continuous tissue cable, promoting a proportion of the axonal sprouts to re-grow and restore function [8], [9]. Recent studies have shown that transplantation of Schwann cells has emerged as a promising therapy for spinal cord repair [12], [15]–[17]. When transplanted into injured spinal cord, Schwann cells enhance axon recovery and provide a growth-supportive substrate for injury axons to regenerate. Interestingly, regenerating axons often accompany with migrating Schwann cells [8]–[10], [17]. To lead axons to regenerate through the injury site, grafted Schwann cells must be able to migrate within injury site and ideally also through the normal tissue.

Several factors have been identified to regulate Schwann cell migration. These factors include neuregulin-1 [18]–[23], brain-derived neurotrophic factor [3], neurotrophin-3 [4] and nerve growth factor [21], [24], [25], extracellular matrix such as laminin [26], [27] and aggrecan [28], and other factors such as Cdc2 [29]. However, these previous studies have focused on the environmental factors regulating Schwann cell migration based primarily on static images or fixed tissues, the intrinsic migratory properties of Schwann cells remain elusive.

In the present study, we established a single-cell migration assay for cultured Schwann cells. This migration assay differs from neuron-glia co-culture assay by absence of neuron-contributed factors and more direct targeting of pharmacological manipulations to Schwann cells. Based on this assay, we examined the intrinsic migratory properties of cultured Schwann cells and the roles of cytoskeletal components during Schwann cell migration.

## Materials and Methods

### Primary Culture and Purification of Schwann Cells

Schwann cells were obtained from sciatic nerves of 2-day-old Sprague-Dawley rat pups and purified using the method described previously [24], [30]. The animal study protocol was approved by the Animal Experimental Committee of Wenzhou Medical College. Briefly, sciatic nerves were separated, demembranated, chopped, and incubated with trypsin (0.25%, Sigma, St Louis, MO) and collagenase (0.03%, Sigma) at 37°C for 20 min. The tissues were then triturated, centrifuged and resuspended in DMEM with 10% heat-inactivated fetal bovine serum (FBS, Hycone, Logan, UT), and plated on 35 mm dishes (Corning) coated with laminin (10 µg/ml) at a density of 5, 000 cells/mm^2^. On the following day, cytosine arabinoside (Ara-C, 1×10^−5^ M, Sigma) was added and left on cells for 2 days. Subsequently, Schwann cells were maintained in DMEM containing 10% FBS, supplemented with forskolin (2 µM) and bFGF (10 µg/ml). The thy 1.1-mediated complement lysis process was repeated every 4–5 days for a total of two passages to reduce contamination of fibroblasts. Cell purity was assessed by labeling cells with S-100 (Sigma) antibody and p-75 NTR (Promega) antibody (Fig. S1). The overall purity of Schwann cells was around 98%.

### Explants Migration Assay

Sciatic nerves explants were described previously [24], [31]. Briefly, sciatic nerves tissues were obtained from 2-day-old Sprague-Dawley rat pups, separated and placed in Leibovitz’s-15 medium (L-15, Gibco, Grand Island, NY), cleared of any blood vessels, musculature and their epineurial sheaths, cut finely with 1 mm diameter. Then the tissues were placed in laminin-coated coverslips (10 µg/ml) with DMEM containing 10% FBS. After incubating at 37°C and 5% CO2 for 48–72 h, coverslips with explants were put into a chamber containing 1 ml serum-free L-15 medium for time-lapse imaging. The chamber was then covered with a thin layer of methyl-siloxane fluid to prevent evaporation. The experiments were carried out at the heated stage (37°C) of a phase contrast microscope (CK40, Olympus Optial, Tokyo, Japan). Images of the migrating Schwann cells were recorded, in a time-lapse mode (one picture every 5 min interval over a total time of 60 min), with a CCD camera (JVC TK-1381, Victor Company, Yokohama, Japan) attached to the microscope, and were stored in a computer for further analysis using Scion imaging software (Frederick, MD). Some pictures were made as movies (Window Movie Marker software), which were presented in the supplementary information.

### Single-cell Migration Assay Based on Time-lapse Imaging

Single-cell migration assay was described previously [32]–[35]. In brief, the purified Schwann cells were re-plated onto square coverslips (8 mm) coated with laminin (10 µg/ml) at a low density of about 1000 cells per coverslip. At 24 h after plating, coverslips with cells were put into a chamber containing 1 ml serum-free L15 medium. The chamber was then covered with a thin layer of methyl-siloxane fluid to prevent evaporation. The experiments were carried out at the heated stage (37°C) of a phase contrast microscope (CK40, Olympus Optial, Tokyo, Japan). Cells with typical morphology of Schwann cells that were not attached to any other cells were selected. Micropipettes used in pulsatile ejection were pulled with a two-stage puller designed for making patch-clamp electrodes. The micropipette with a tip opening of about 1 µm was placed 15 µm perpendicular and 100 µm away from the center of cell under test. A standard pressure pulse of 3 psi (1 psi = 6.89 kPa) in amplitude and 20 ms in duration was generated by a pulse generator and applied to the pipette at a frequency of 2 Hz. Under this standard condition, the concentration of factor at 100 µm from pipette tip was about 10^−3^ fold lower than that in the pipette [36]. Images of the migrating Schwann cells were recorded, in a time-lapse mode (one picture every 5 min interval), with a CCD camera (JVC TK-1381, Japan) attached to the microscope, and were then stored in a computer for further analysis using Scion imaging software (Frederick, MD). Briefly,we measured the distance of cell migration during a control period and after treatment, and calculated the respective migration rates (distance/time).

### Immunocytochemistry

In brief, the purified Schwann cells and sciatic nerves explants were fixed with fresh 4% paraformaldehyde in 0.1 M PBS (pH 7.4) for 20 min. After washing with PBS, cells were permeabilized with 0.2% Triton X-100 in 0.1 M PBS for 5 min, followed by incubation in blocking buffer (5% normal goat serum and 0.2% Triton X-100 in 0.1 M PBS, pH 7.4) for 1 h, and incubated overnight at 4°C with a polyclonal antibody against p-75 (1∶500, Promega, Madison, WI); with monoclonal anti-S-100 antibodies (1∶500, Sigma) or acetylated tubulin (1∶1000, Sigma) or p-MLC(1∶50, Cell Signaling Technology) diluted in the blocking buffer. Cells were washed three times with PBS and incubated for 1 h at room temperature with an appropriate fluorescence-conjugated secondary antibody (1∶1000, Molecular probe, Eugene, OR), and then visualized under confocal fluorescence microscopy (FV1000, Olympus). No positive signal was observed in control incubations using no primary antibody. For visualization of F-actin, cells were incubated with rhodamine-conjugated phalloidin (1∶60, Molecular probe) at room temperature for 1 h.

For Dil label, Schwann cells were fixed with fresh 4% paraformaldehyde in 0.1 M PBS (pH 7.4) for 20 min. After washing with PBS, cells were incubated for 30 min at room temperature with Dil dye (10 µM, Molecular probe). Washing with PBS three times, cells were then visualized under confocal fluorescence microscopy (FV1000, Olympus).

### Sources and Preparation of Reagents

2, 3-butanedione monoxime (BDM), Latrunculin A (LA), Cytochalasin D (CD), and ML-7 were from Sigma-Aldrich. Jasplakinolide (Jasp) was from Invitrogen, and Caly was from Millipore, and Y-27632 was from Calbiochem. Pharmacological agents were dissolved in DMSO or PBS in stock solution. 50 µM LA, or 1 mM CD or 20 µM Jasp or 5 mM ML-7 or 200 mM BDM or 25 µM Caly or 10 mM Y-27632 was loaded into the micropipette for migration assay.

### Statistical Analysis

All data presented represent results from at least three independent experiments. Statistical analysis was performed by using one-way ANOVA with pair-wise comparisons by the Student-Newman-Keuls method. Statistical significance was defined as *P*<0.05.

## Results

### Migratory Properties of Schwann Cells from Sciatic Nerve Explants

**Figure 1:**
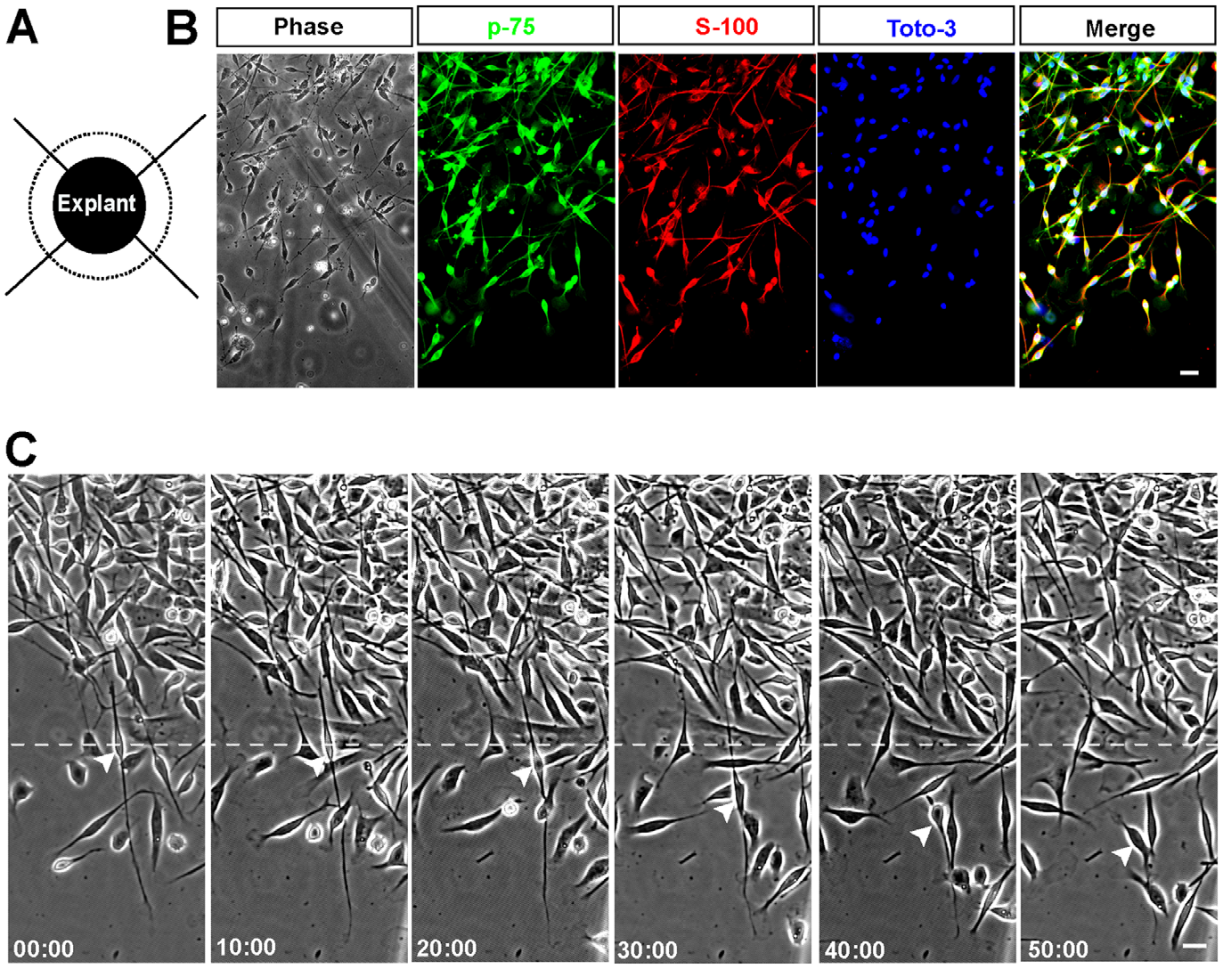
Spontaneous migration of Schwann cells from sciatic nerve explants. (**A**) A model for sciatic nerve explants. (**B**) Identification of migrating Schwann cells away from sciatic nerve tissues by immunostaining. P-75 (green) and S-100 (red) are cell markers of Schwann cells. Toto-3 (blue) is labeled for nucleus. (**C**) Time-lapse images of migrating Schwann cells from sciatic nerve explants. White arrowheads indicated for example cell. Time, min; scale bars, 20 µm.

To explore migratory properties of Schwann cells, the sciatic nerve explants migration assay was firstly performed (Fig. 1A). The sciatic nerve tissues were cultured on the cover-slips coated with laminin, then the cells could spontaneously migrate away from tissues after 48–72 h. As shown in Fig. 1B, most of the spontaneous migrating cells from explants displayed bipolar morphology and the double immuno-staining showed that (83.6±10.6) % of these cells external to the explants (n = 15) were S-100 and p-75 positive, suggesting that most of cells migrating out from the sciatic nerve explants were Schwann cells, and a few of cells were other unidentified cells.

The explants were observed under the microscope based on time-lapse imaging. As shown in Fig. 1C and Movie S1, Schwann cells from sciatic nerve tissues displayed high motility. There was one major mode of Schwann cell migration away from sciatic nerve tissues. One Schwann cell would send out one long process, giving it a bipolar fusiform morphology (Fig. 1C, cell indicated by white arrowhead, as an example). The nucleus of this cell then moved from one end of the process to the other newer end, with a distance farther than 100 µm. The back part of the process would retract, even disappear and the front part would move ahead. The soma then migrated based on the front process adherence. This migration mode mainly was mediated by the front process. Most of Schwann cells migrating from sciatic nerve tissues adopted this mode.

### Schwann Cells Displayed Three Motile Phenotypes in Single-cell Migration Assay

To further examine migratory properties of Schwann cells, cultured Schwann cells were purified. Primary cultured Schwann cells mainly displayed three morphological phenotypes, uniploar (9.5±1.2%), bipolar (79.9±1.6%) and multipolar shape (10.6±1.4%) (Fig. S1A,C). The purity of Schwann cells was identified by double immuno-staining of p-75 and S-100 (Schwann cell markers). As shown in Fig. S1B, more than 98% of purified Schwann cells were immuno-positive for S-100 and p-75.

**Figure 2:**
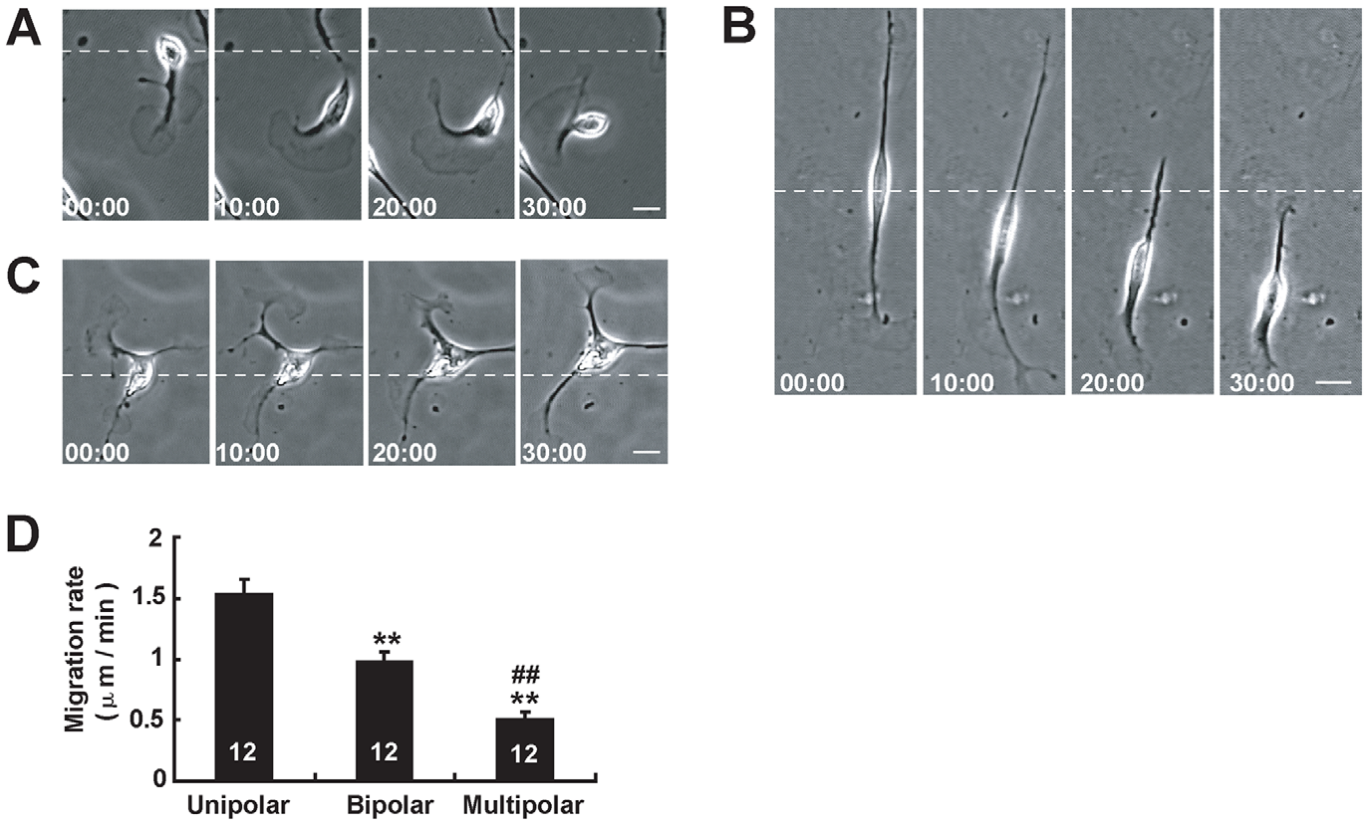
Cultured Schwann cells displayed distinct motile phenotypes in single-cell migration assay. (**A–C**) Time-lapse images of Schwann cells with distinct motile morphologies: unipolar (**A**), bipolar (**B**) and multipolar (**C**). (**D**) Histogram showing the average migration rates of each motile phenotype of Schwann cells. Time, min, scale bar, 20 µm. Data are mean ± sem. **P<0.01, significant difference compared with unipolar group, ^##^ *P*<0.01, significant difference compared with bipolar group, one-way ANOVA with pairwise posthoc tests comparisons by Student-Newman-Keuls method.

We next established a single-cell migration assay to examine the intrinsic migratory properties of Schwann cells. Purified Schwann cells were re-planted on the cover-slips coated with laminin at a low density of 1000 cells per coverslip. Under the microscope, cells with typical Schwann cell morphology were selected, which had no contact with any other cells. Based on time-lapse imaging of migratory behavior of single isolated Schwann cell, we found that cultured Schwann cells mainly displayed three motile phenotypes, uniploar, bipolar and multipolar phenotypes (Fig. 2), and each phenotype adopted different migration mode.

The first mode of migration was adopted by unipolar phenotype of Schwann cells. As shown in Fig. 2A, selected Schwann cell had a unipolar shape, one dynamic leading front and a soma. The leading front usually had one large lamellipodium and moved forward, with the soma following at the rear. The entire cell moved as a unit. The second mode of migration was adopted by the bipolar phenotype of Schwann cells. As shown in Fig. 2B, selected cell had a bipolar shape, and sent out two processes during migration. One process with larger lamellipodia and/or higher dynamic filopodia in the direction of cell migration was considered as ‘leading process’, the other process in the opposite direction that had smaller lamellipodia and/or less dynamic filopodia was considered as ‘trailing process’. As the leading process moved ahead, the nucleus of this cell moved forwards simultaneously. Meanwhile, the trailing process underwent gradual retraction. Most of cultured Schwann cells were bipolar morphology, thus Schwann cells mainly adopted this migration mode.

The third mode of migration was adopted by multipolar phenotype of Schwann cells. As shown in Fig. 2C, the selected cell with a multipolar shape, spent the majority of their time moving the tips of their extensions as the soma moved slowly. To quantify the motility of each motile phenotype, we measured migration rates of Schwann cells. Statistical analysis revealed that unipolar phenotypes moved fastest, and bipolar phenotypes had higher motility than multipolar phenotypes (Fig. 2D). These results suggest that cultured Schwann cells display malleable motile phenotypes.

### Motile Phenotypes of Schwann Cells Could Spontaneously Transform into Each Other

**Figure 3:**
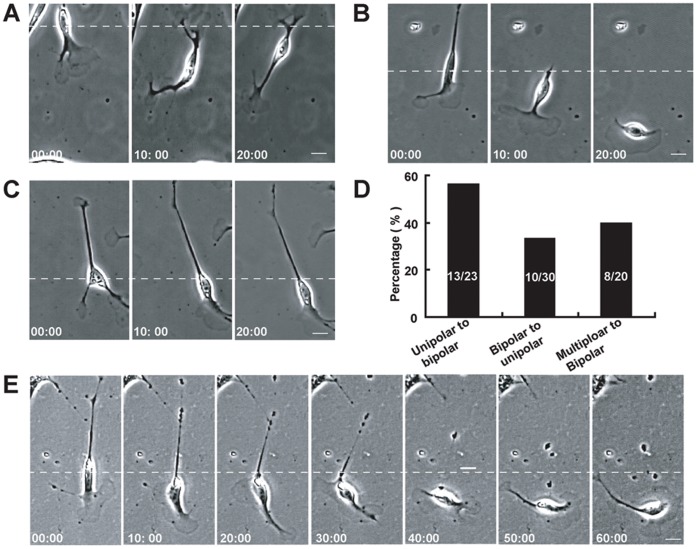
Distinct motile phenotypes of Schwann cells could transform into each other spontaneously. The example cells were shown in a series of time-lapse images (**A–C**). (**A**) A Schwann cell with unipolar morphology gradually transformed into a bipolar phenotype. (**B**) A Schwann cell with bipolar morphology transformed into a unipolar phenotype. (**C**) A Schwann cell with multipolar morphology rapidly transformed into a bipolar phenotype. (**D**) Histogram showing the percentages of transformed Schwann cells in total observed cells. (**E**) A sample cell showing that one Schwann cell changed its direction of migration through morphological transformation. Time, min, scale bar, 20 µm.

In the course of time-lapse imaging, we found that these different motile phenotypes of Schwann cells could spontaneously transform into each other. As shown in Fig. 3A, one Schwann cell displayed a unipolar morphology at the start of the time-lapse recording, rapidly transformed into a bipolar phenotype. Statistical analysis revealed that 56.5% of 23 Schwann cells with unipolar morphology transformed into Schwann cells with bipolar morphology in half an hour (Fig. 3D). Meanwhile, we also observed that one Schwann cell with a bipolar morphology also rapidly transformed into a unipolar phenotype. As shown in Fig. 3B, the leading process of this cell moved forward accompanied by enlarged lamellipodia, while there was a gradual retraction of the trailing process. At 20 min, the trailing process completely retracted and disappeared, and the leading process became a large lamellipodium (Fig. 3B). The entire cell became a fan-like morphology. Quantitative analysis revealed that 33.3% of 30 Schwann cells with bipolar morphology transformed into Schwann cells with unipolar morphology in half an hour (Fig. 3D). Interestingly, we also observed that one Schwann cell with multipolar morphology also could rapidly transform into a bipolar phenotype (Fig. 3C). Quantitative analysis revealed that 40% of 20 Schwann cells with multipolar morphology transformed into Schwann cells with bipolar morphology in half an hour (Fig. 3D). These results suggest that different motile phenotypes of Schwann cells can spontaneously transform into each other. Interestingly, during spontaneous migration, some Schwann cells could change spontaneously from the bipolar to the unipolar morphology, and then rapidly changed back. The direction of migration was also changed (Fig. 3E). These results suggest that Schwann cells can change the direction of migration through morphological transformation.

### F-actin Polymerization at Leading Front was Required for the Extension of Leading Process and Soma Translocation of Schwann Cells

**Figure 4:**
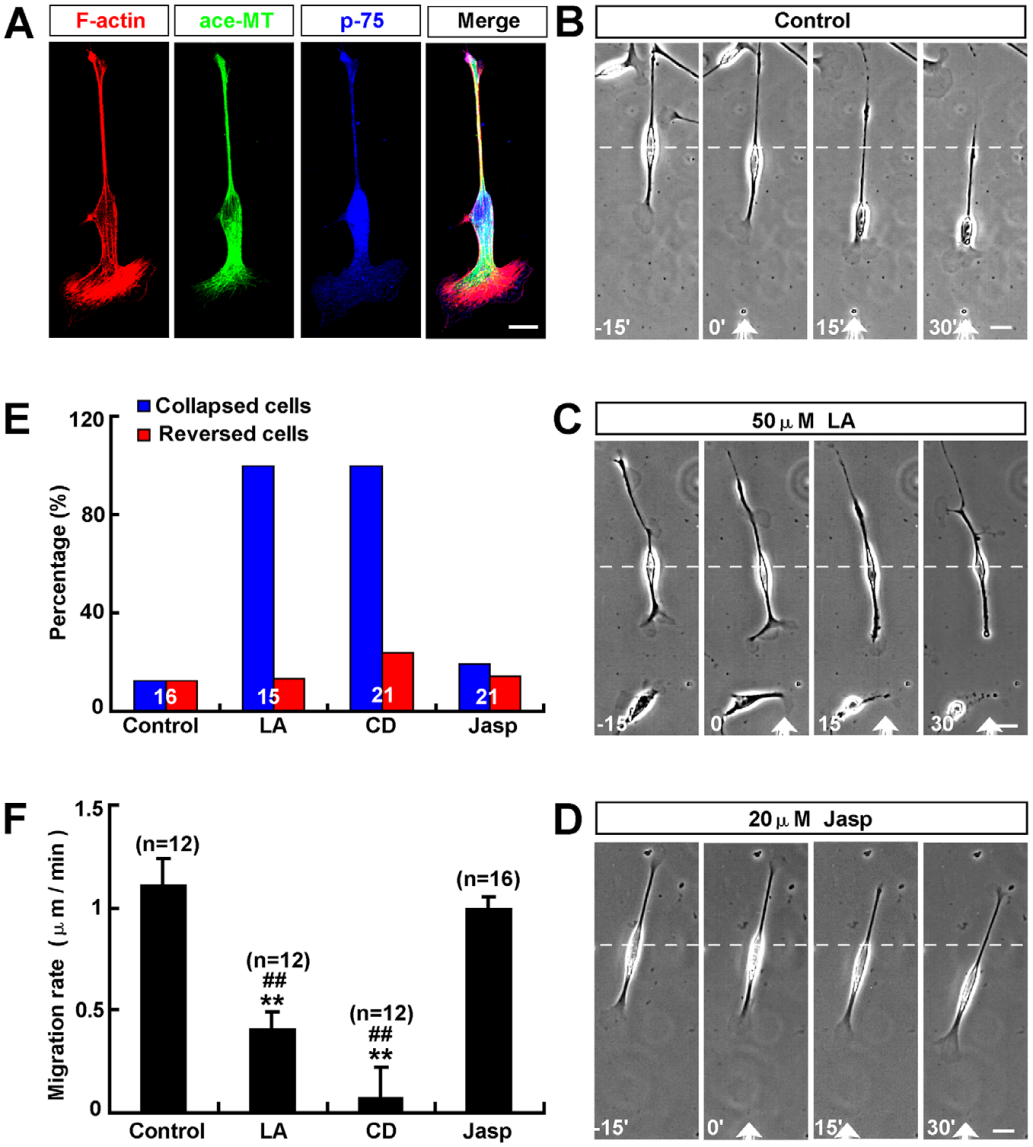
F-actin polymerization at the leading front was required for extension of leading process and soma translocation of Schwann cells. (**A**) Distributions of F-actin (red) and microtubules (green) in Schwann cells by immunostaining. P-75 (blue) is a cell marker of Schwann cell. (**B–D**) Images of migrating Schwann cells before and after frontal application of a DMSO, as a control (**B**), or latrunculin A (LA, **C**) or Jasp (**D**) gradient. (**E–F**) Summary of the percentages of collapsed or reversed cells in total observed cells (**E**) and migration rates of soma (**F**) after frontal application of DMSO or LA (50 µM in the pipette) or CD (1 mM in the pipette) or Jasp (20 µM in pipette).White arrowheads indicated the direction of the micropipette. Time, min; scale bars, 20 µm. Data are mean ± s.e.m. **P<0.01, significant difference compared with control, ^##^ *P*<0.01, significant difference compared with Jasp group, one-way ANOVA with pairwise posthoc tests comparisons by Student-Newman-Keuls method.

Given that most of cultured Schwann cells exhibited bipolar morphology (Fig. S1A,C), we next examined the molecular mechanisms of these motile phenotypes during migration. Since the reorganization of cytoskeleton in leading front play a key role in cell migration [37], [38], we first examined the roles of cytoskeleton in migrating Schwann cells. To visualize F-actin and microtubules, Schwann cells were fixed and simultaneously labeled for F-actin using rhodamine-conjugated phalloidin and microtubules with specific antibody against acetylated a-tubulin, a marker for stable microtubules. As shown in Fig. 4A, in the Schwann cells, actin filaments were predominant in the central domain of lamellipodia and some of F-actins were distributed at the edge of lamllipodia, whereas bundles of microtubules were localized to the central domain and few free microtubules penetrated into the peripheral domain. These results suggest that F-actin may play a key role in the migration of Schwann cells.

To further examine the roles of F-actin in Schwann cell migration, we used latrunculin A (LA) or cytochalasin D (CD) (F-actin polymerization inhibitors) drug to inhibit F-actin polymerization during migration. We used a micropipette assay to create drug concentration gradient. This assay has been used in our previous studies (Huang et al., 2008; Huang et al., 2011a; Huang et al., 2011b, See material and methods). In this assay, the closer to the micropipette, the higher concentration of drugs was formed. A gradient of LA or CD was produced in front of the isolated migrating Schwann cells by using a micropipette loaded with 50 µM LA or 1 mM CD with repetitive injection by air pressure. As shown in Fig. 4C, after frontal application of a LA or CD gradient, the leading front was inhibited in their motility and showed collapse and retraction within 15 min. After half an hour, the leading process totally collapsed and retracted. The motility of soma was dramatically inhibited. However, the trailing process was not affected. In few cases, the soma later reversed their direction of translocation, with the original trailing tail becoming a new leading process. Schwann cell migration was not affected by a gradient of Jasplakinolide (Jasp), which stabilizes F-actin, or DMSO as a control (Fig. 4B,D). Quantitative analysis revealed that 12.5% of 16 (19.1% of 21 under Jasp gradient) Schwann cells collapsed and 12.5% of 16 (14.3% of 21 under Jasp gradient) Schwann cells reversed soma translocation under DMSO gradient, whereas, 100% of 15 (100% of 21 under CD gradient) Schwann cells collapsed and 7% of 15 (23.8% of 21 under CD gradient) Schwann cells reversed soma translocation under LA gradient (Fig. 4E). The average migration rate of soma significantly was decreased under LA or CD gradient, not significantly changed under Jasp gradient, compared to control (Fig. 4F). Quantitative analysis also revealed that average migration rates of soma had significant differences between LA or CD and Jasp group. Taken together, these results suggest that F-actin polymerization is required for the extension of leading front and soma translocation of Schwann cells.

### The Polarized Distribution of Myosin II Activity was Required for Soma Translocation of Schwann Cells

**Figure 5:**
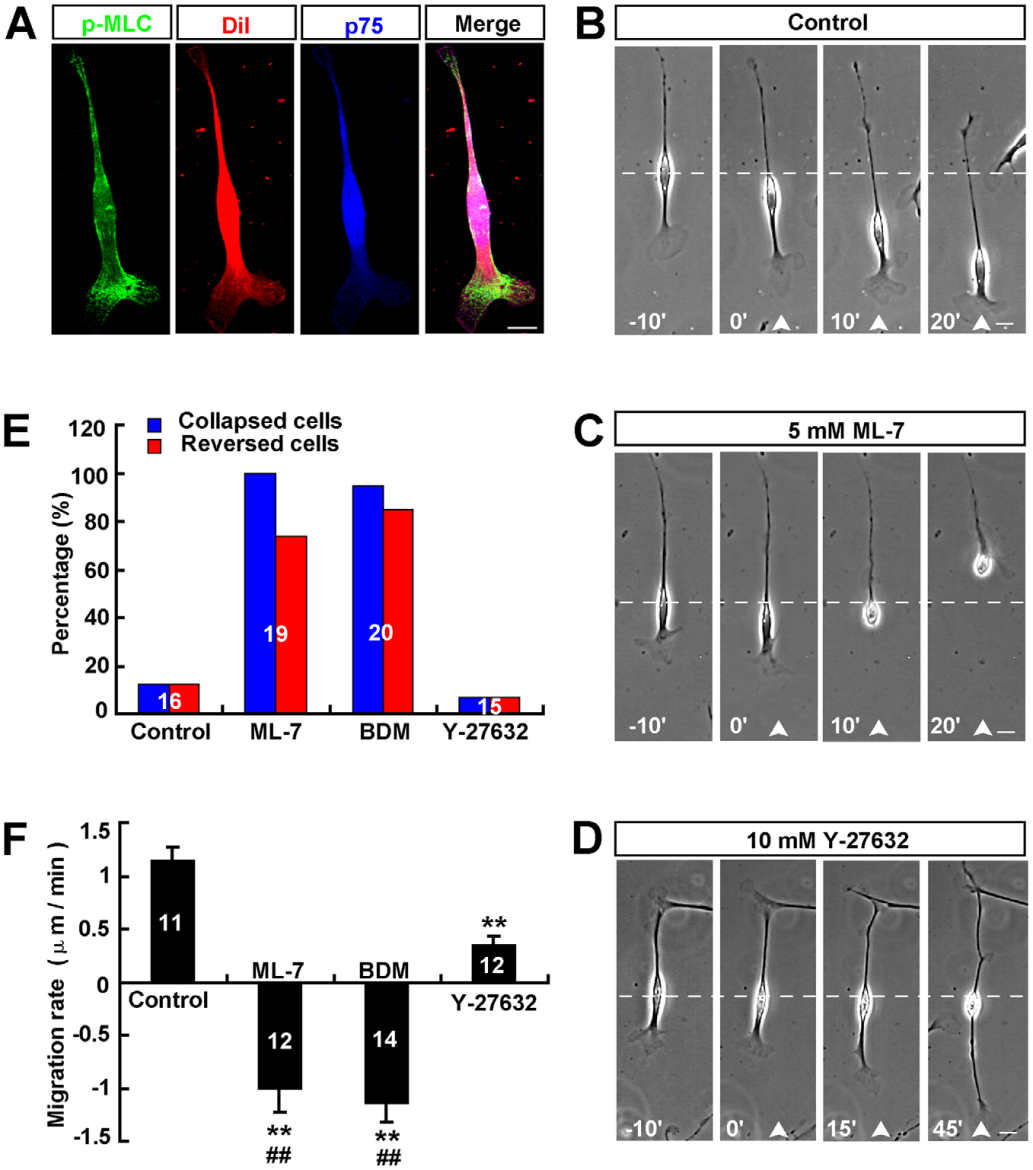
Decreasing the front-to-rear difference of myosin II activity induced the collapse of leading front and reversed soma translocation of Schwann cells. (**A**) Distributions of p-MLC (green) and Dil (red, a marker of membrane) in Schwann cells by immunostaining. P-75 (blue) is a cell marker of Schwann cell. (**B–D**) Images of migrating Schwann cells before and after frontal application of a DMSO (**B**), or ML-7 (**C**) or Y-27632 (**D**) gradient. (**E–F**) Summary of the percentages of collapsed or reversed cells in total observed cells (**E**) and migration rates of soma (**F**) after frontal application of a DMSO or ML-7(5 mM in the pipette) or BDM (200 mM in the pipette) or Y27632 (10 mM in the pipette) gradient. White arrowheads indicated the direction of the micropipette. Time, min; scale bars, 20 µm. Data are mean ± s.e.m. **P<0.01, significant difference compared with control, ^##^ *P*<0.01, significant difference compared with Y-27632 group, one-way ANOVA with pairwise posthoc tests comparisons by Student-Newman-Keuls method.

In migrating neurons, the leading process pulls soma forward through myosin II-dependent forward F-actin flow [39], thus we next examined whether myosin II was also involved in soma translocation of Schwann cells. We first observed the distribution of p-MLC (myosin light chain, MLC) in Schwann cells by immunostaining, which marks the activated form of myosin II. We labeled the plasma membrane by using membrane marker Dil dye (1, 1′–dioctadecyl–3, 3, 3′, 3′–tetramethylindocarbocyanine) to observe the relative distribution of p-MLC in Schwann cells. As shown in Fig. 5A, p-MLC displayed a polarized distribution in Schwann cells, with the leading process exhibiting higher than the soma and trailing process.

To further examine whether myosin II is involved in directional migration of Schwann cells, we applied a gradient of ML-7 (5 mM in micropipette), a specific inhibitor of myosin light chain kinase, or BDM (200 mM in micropipette), an inhibitor of myosin II, in front of migrating Schwann cell to disrupt the polarized distribution of myosin II activity across cell. As shown in Fig. 5C, after frontal application of ML-7 or BDM gradient, the leading front was inhibited in their motility and showed collapse and retraction within 10 min. Most of the tested cells, soma later reversed their direction of translocation, with the original trailing tail becoming a new leading front (Fig. 5C). Surprisingly, different from ML-7, or BDM gradient, as shown in Fig. 5D, the Rho kinase (an upstream activator of myosin II) inhibitor Y-27632 gradient dramatically promoted the extension of leading process, but reduced soma translocation. Schwann cell migration was not affected by a DMSO gradient, as a control (Fig. 5B). Quantitative analysis revealed that 12.5% of 16 (6.7% of 15 under Y27632 gradient) Schwann cells collapsed and 12.5% of 16 (6.7% of 15 under Y27632 gradient) Schwann cells reversed soma translocation under DMSO gradient, whereas, 100% of 19 (95% of 20 under BDM gradient) Schwann cells collapsed and 74% of 19 (85% of 20 under BDM gradient) Schwann cells reversed soma translocation under ML-7 gradient (Fig. 5E). The average migration rate of soma significantly was decreased under ML-7 or BDM or Y27632 gradient, compared to control (Fig. 5F). Quantitative analysis also revealed that the average migration rates of soma had significant differences between ML-7 or BDM and Y-27632 (Fig. 5F). These results suggest that the polarized distribution of myosin II may mediate soma translocation of Schwann cells.

**Figure 6:**
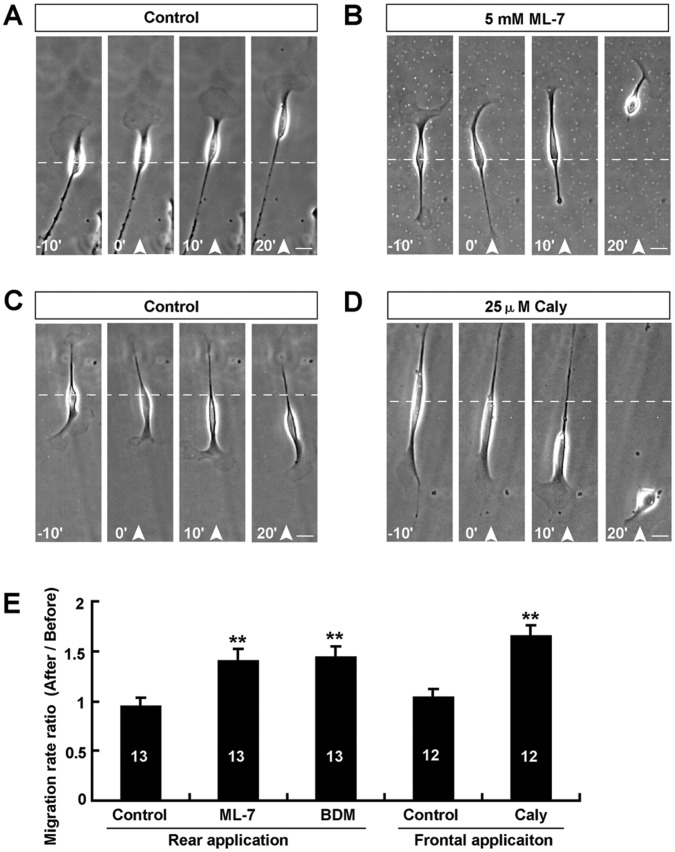
Increasing the front-to-rear difference of myosin II activity accelerated soma translocation of Schwann cells. (**A–B**) Images of migrating Schwann cells before and after rear application of a DMSO (**A**), or ML-7 (**B**) gradient. (**C–D**) Images of migrating Schwann cells before and after frontal application of a DMSO (**C**), or Caly (**D**) gradient. (**E**) Summary of migration rate ratios (after/before) of soma under various conditions. White arrowheads indicated the direction of the micropipette. The micropipette was loaded with 5 mM ML-7, or 200 mM BDM, or 25 µM Caly. Time, min; scale bars, 20 µm. Data are mean ± s.e.m. For data from rear application, **P<0.01, significant difference compared with control, one-way ANOVA with pairwise posthoc tests comparisons by Student-Newman-Keuls method. For data from frontal application, **P<0.01, significant difference compared with control, Student's *t*-test.

We next examined whether increasing the front-to-rear difference of myosin II activity could promote soma translocation of Schwann cells. We applied the ML-7 or BDM gradient from the rear to decrease of myosin II activity at the trailing process. As shown in Fig. 6B, after rear application of a ML-7 or BDM gradient, the trailing process collapsed and retraction, whereas, soma translocation was accelerated dramatically. Schwann cell migration was not affected by a DMSO gradient, as a control (Fig. 6A). Quantitative analysis revealed that the average migration rate ratio significantly was increased under ML-7 or BDM gradient, compared to control (Fig. 6E). These results suggest that the front-to-rear difference in myosin II activity may mediate soma translocation of Schwann cells. To further support this notion, as shown in Fig. 6D,E, after frontal application of the phosphatase inhibitor Caly (25 uM in micropipette), which activates myosin II activity by keeping the myosin regulatory light chain phosphorylated, soma translocation was also accelerated dramatically. Taken together, these results strongly suggest that polarized distribution of myosin II activity mediates soma translocation of Schwann cells during migration.

## Discussion

Schwann cell migration is a key process during axon myelination and is also essential for regeneration and remyelination after nerve injury [6], [9], [10]. Most previous studies relied on bath treatment of pharmacological agents or over-expression or knockout of proteins to examine the molecular mechanisms underlying Schwann cell migration. However, these manipulations will interfere with signaling molecules either globally or chronically. In the present study, we used local perfusion of pharmacological agents (this assay has been successfully used in our previous studies [32], [33], [35]) to single-isolated migrating Schwann cells to examine the roles of cytoskeletal components during Schwann cell migration. We found that during migration, Schwann cell displayed malleable motile phenotypes and extension of leading front dependent on F-actin polymerization pulled soma forward translocation mediated by myosin II activity.

In our studies, laminin was chosen as a substrate for Schwann cell migration as it is expressed in static nerve and can affect motility and morphology of Schwann cells [26], [40]. On the laminin substrate, we found that Schwann cells displayed distinct motile phenotypes. Schwann cells with unipolar or bipolar shape have higher motility than the multipolar shape. Our results were consistent with one previous study, which showed that Schwann cells cultured on the engineered biomaterials also displayed distinct motile phenotypes [41]. In order to migrate, cells must be polarized. They usually exhibit a polarized morphology, with a highly dynamic leading front and a thin trailing tail [42], [43]. Consistent with the migratory behavior of most general cells, unipolar and bipolar shape of Schwann cells are polarized morphology, thus they have higher motility. Interestingly, these distinct motile phenotypes could spontaneously transform into each other. Because most of Schwann cells display bipolar shape, we propose that unipolar or multipolar shape may be transient phenotypes of bipolar shape. To support this notion, we found that some Schwann cells with bipolar shape could rapidly transform into the unipolar, then transform back into the bipolar, and the direction of cell migration was also changed (Fig. 3E). These results suggest that Schwann cells can change their direction of migration through morphological transformation. The migratory properties of Schwann cells *in vitro* may indicate their functional properties *in vivo*. During development or peripheral nerve injury, Schwann cells may establish a bipolar polarized morphology, and migrate along the axon. Either spontaneously or stimulated by some extracellular factors, some of these Schwann cells may change their morphology and stop to ensheath and myelinate the axon or change their direction of migration.

Recent studies have identified several specific effectors that modulate actin reorganization in myelinating Schwann cells. These effectors include myosin II [5], neural Wiskott-Aldrich syndrome protein (N-WASP) [44], [45] and Rho-GTPase family [46]–[50]. These previous studies have focused on the roles of actin dynamics in morphology and differentiation of Schwann cells, and interactions between Schwann cells and axons, however, the roles of actin dynamics in migration of Schwann cell during myelination remain elusive. We found that F-actin cytoskeleltal proteins mainly distributed at leading front of migrating Schwann cells. Local disruption of F-actin polymerization at leading front by LA or CD induced the collapse and retraction of leading front, and inhibited soma translocation. These results suggest that F-actin polymerization at leading front is essential for extension of leading process and soma translocation of Schwann cells during migration. Schwann cell migration is a key process during axon myelination [6], [9], [10]. Previous studies have shown that disruption of actin polymerization by CD inhibits myelination of Schwann cells through affecting the expression of myelin-specific genes [51]. Our studies may provide another possibility that actin polymerization drug CD may inhibit myelination of Schwann cells partially through inhibiting their motility.

Soma translocation of Schwann cells implicates the progression of the inner (axon-related) Schwann cell process during myelination [52]. Myosin II has been identified as a key player in soma translocation in many types of cells such as neurons [39], [53]–[55]. It is still unknown whether myosin II is also involved in regulating soma translocation of Schwann cells. Recent studies have reported that in cultured cerebellar granule cells, the activity of myosin II is enriched in the proximal region of leading process, and is responsible for the coordinated motility of centrosome and soma [55], generating traction force along the leading process that drives forward translocation of soma [39]. Consistent with these findings, we found that myosin II activity displayed a polarized distribution with the leading process expressing higher than soma in migrating Schwann cells. Frontal application of myosin II inhibitor to migrating neuron only suppresses soma translocation, but not affect the leading front [39]. However, in the present study, in migrating Schwann cells, inhibition of myosin II activity of leading front by ML-7 or BDM gradient induced the collapse and retraction of leading front, and reversed soma translocation. One previous study has shown that ML-7 induced the retraction of mature oligodendrocyte processes [56]. These results suggest that different functions of myosin II exist in neuron and glia cells. Interestingly, the collapse of leading front by LA or CD only inhibited soma translocation, whereas the collapse of leading front by ML-7 or BDM could reverse soma translocation. These results suggest that myosin II is not only involved in the lamellipodia extension of leading front, but also is essential for re-orientation and stabilization of the direction of soma translocation in Schwann cells. To further support this notion, we found that decreasing or increasing the front-to-rear difference of myosin II activity reversed or accelerated soma translocation (Fig. 5 and Fig. 6). Thus, we proposed that the polarized distribution of myosin II activity is required for the extension of leading process and soma translocation of Schwann cells. Interestingly, one previous study has shown that myosin II regulates the peripheral myelin formation [5]. Schwann cells lacking myosin II activity exhibit a multipolar morphology, which fail to elongate properly along the axon and myelinate [5]. Our studies may provide another possibility that myosin II regulates the peripheral myelin formation partially through mediating soma translocation.

Myosin II is known to be activated by Rho kinase [57]. Previous studies have shown that inhibition of ROCK-mediated MLC phosphorylation promotes the coordinated wrapping of axon [48], whereas inhibition of myosin II ATPase activity or down-regulation of myosin II levels blocked myelin formation [5]. Consistent with these findings, we found that Y-27632 gradient (Rho kinase inhibitor) promoted the extension of process and inhibited soma translocation, whereas ML-7 or BDM gradient induced the collapse of leading front and reversed soma translocation. These contrasting effects provide compelling evidence for existence of different mechanisms controlling myosin II activity in Schwann cells. In fact, in other cell types such as fibroblast cells, MLC and Rho kinase spatially regulate the phosphorylation of Myosin II. Myosin II activated by MLC kinase at the cell periphery controls membrane ruffling, whereas Myosin II activated by Rho kinase at the cell center regulates the central stress fiber system and focal adhesion formation [58], [59]. Thus, we propose that the different spatial regulation of Myosin II activity by MLC and Rho kinase may determine the different responses to their inhibitors during Schwann cell migration. Further studies should be carried out to test these possibilities.

In conclusion, we established a single-cell migration assay for Schwann cells based on time-lapse imaging, and showed that during migration, Schwann cells displayed malleable motile phenotypes and the extension of leading front dependent on F-actin polymerization pulled soma forward translocation mediated by myosin II activity. This knowledge will be helpful for us to better understand the mechanism of Schwann cell migration during development of the peripheral nervous system and regeneration and remyelination after nerve injury.

## Supporting Information

Figure S1 **Cultured Schwann cells mainly displayed three morphological phenotypes.** (**A**) Schwann cell phases in primary cultures. (**B**) Identification of cultured Schwann cells by immunostaining. P-75 (green) and S-100 (red) are cell markers of Schwann cells. Toto-3 (blue) is labeled for nucleus. White arrowhead, double arrowhead and arrow indicated unipolar, bipolar and multipolar shape of Schwann cells, respectively. (**C**) Histogram showing the average percentages of each morphological phenotype in one field (n = 40). Data are mean ± SD. Scale bars, 20 µm.
(TIF)

Movie S1 **Spontaneous migration of Schwann cells from sciatic nerve explants based on time-lapse imaging.** Time, min.
(WMV)
