# Supplementary figures and images for: Intrinsic Migratory Properties of Cultured Schwann Cells Based on Single-Cell Migration Assay
Source: PLoS One. 2012 Dec 14;7(12):e51824. doi: 10.1371/journal.pone.0051824 (PMC3522601; doi:10.1371/journal.pone.0051824)

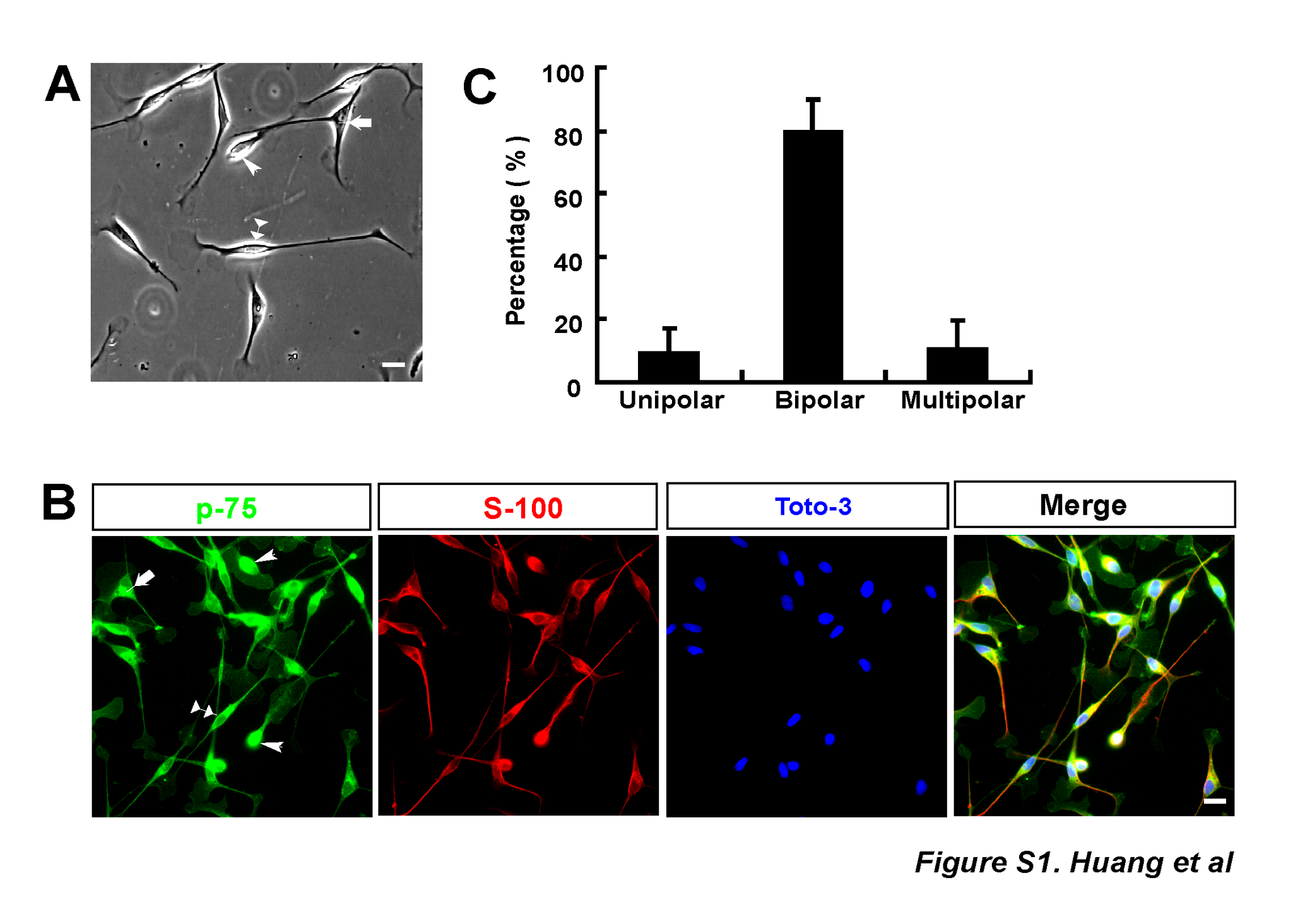

Supplement: Figure S1 — Cultured Schwann cells mainly displayed three morphological phenotypes. (A) Schwann cell phases in primary cultures. (B) Identification of cultured Schwann cells by immunostaining. P-75 (green) and S-100 (red) are cell markers of Schwann cells. Toto-3 (blue) is labeled for nucleus. White arrowhead, double arrowhead and arrow indicated unipolar, bipolar and multipolar shape of Schwann cells, respectively. (C) Histogram showing the average percentages of each morphological phenotype in one field (n = 40). Data are mean ± SD. Scale bars, 20 µm. (TIF) [file pone.0051824.s001.tif]
